# Postoperative Complications after Thoracic Surgery in the Morbidly Obese Patient

**DOI:** 10.1155/2011/865634

**Published:** 2011-12-28

**Authors:** Lebron Cooper

**Affiliations:** University of Miami Miller School of Medicine, Miami, FL 33136, USA

## Abstract

Little has been recently published about specific postoperative complications following thoracic surgery in the morbidly obese patient. Greater numbers of patients who are obese, morbidly obese, or supermorbidly obese are undergoing surgical procedures. Postoperative complications after thoracic surgery in these patients that can lead to increased morbidity and mortality, prolonged hospital stay, and increased cost of care are considered. Complications include difficulties with mask ventilation and securing the airway, obstructive sleep apnea with risk of oversedation, pulmonary complications related to reduced total lung capacity, reduced functional residual capacity, and reduced vital capacity, risks of aspiration pneumonitis and ventilator-associated pneumonia, cardiomyopathies, and atrial fibrillation, inadequate diabetes management, positioning injuries, increased risk of venous thrombosis, and pulmonary embolism. The type of thoracic surgical procedure may also pose other problems to consider during the postoperative period. Obese patients undergoing thoracic surgery pose a challenge to those caring for them. Those working with these patients must understand how to recognize, prevent, and manage these postoperative complications.

## 1. Introduction

Little has been recently published about specific postoperative complications following thoracic surgery in the morbidly obese patient. Anesthesia and postoperative management of morbidly obese patients in thoracic surgery are based on experience in these patients undergoing other types of procedures [1]. While approximately 5% of patients undergoing surgical procedures are considered morbidly obese (BMI > 40 kg/m^2^), another 30% of patients in the US are considered obese (BMI > 30 kg/m^2^) [2]. The exact number of these who require thoracic surgery is unknown. However, considering that postoperative complications are a major cause of morbidity, mortality, prolonged hospital stay, and increased cost of care, it is important that those working with these patients during the postoperative period understand how to recognize, prevent, and manage these complications [3]. 

## 2. Airway Complications

Mask ventilation and intubation may be difficult in the morbidly obese patient secondary to excessive tissue in the posterior pharyngeal wall [4]. A Mallampati score of III or IV and increased neck circumference have been found to be the best predictors of potential difficulty with tracheal intubation [5]. These considerations should be kept in mind when planning extubation following thoracic surgery in the morbidly obese patient, whether using a double-lumen tube or a single-lumen endotracheal tube with a bronchial blocker. The extubation plan should consider the initial ease of mask ventilation, the difficulty of intubation (should reintubation become necessary), and the type of procedure performed. In a comparison between transmediastinal and transthoracic esophagectomy, Bartels et al. found that early extubation (within 6 hours) following transthoracic esophagectomy prolongs ICU length of stay and leads to an increase rate of mortality [6], although the recent trend to extubate immediately after resection in the operating room has been shown to be equally safe. When comparing early extubation in the operating room to late extubation in the ICU, Lanuti et al. found that operative approach did not influence the failure to extubate [7]. Positioning morbidly obese patients in reverse Trendelenburg has been suggested to optimize ventilation and access to the airway should the need for reintubation occur [1]. 

## 3. Obstructive Sleep Apnea

Although most morbidly obese patients have probably not had previous sleep studies to prove the existence of obstructive sleep apnea, many may actually have this disease. The American Society of Anesthesiologists Task Force on Perioperative Management Practice Guidelines for the perioperative management of patients with obstructive sleep apnea warns that judicious use of sedatives and opiates in the perioperative period is indicated in patients with obstructive sleep apnea [8]. It is probably wise to consider judicious use of sedatives and opiates in all morbidly obese patients to prevent oversedation and delayed airway obstruction. Nonsteroidal analgesics may reduce the need for opiates in the postoperative period [9]. 

## 4. Pulmonary Complications

Morbidly obese patients have reduced total lung capacity, reduced functional residual capacity, and reduced vital capacity [10]. Alveolar arterial oxygenation gradient is increased, and atelectasis has been found to persist for at least 24 hours in morbidly obese patients, whereas it disappeared in the nonobese [11]. Consideration should be given to the increased likelihood of pulmonary complications in these patients postoperatively. Complications related to residual atelectasis may result in desaturation. Pneumonia, bronchospasm, atelectasis, acute respiratory insufficiency, prolonged ventilation, and bronchial infections were found in 33.9% of patients with mild to moderate COPD undergoing general surgery. Risk factors for increased pulmonary complications were male gender, amount of smoking, duration of surgery over 270 minutes, low FEV1/FVC ratio, and chest or upper abdominal incision [12]. A study of 147 lobectomies, comparing VATS versus open thoracotomy, found that VATS patients, in spite of having more comorbidities, had significantly less postoperative pneumonia, fewer chest tube days, and a shorter hospital length of stay [13]. 

Ventilator-associated pneumonia can be a life-threatening complication in the course of 8–28% of mechanically ventilated patients. Mortality rates range between 24 to 50% and can be as high as 76%, when lung infection is with high-risk pathogens [14]. Several studies have shown that prompt diagnosis and appropriate antimicrobial treatment result in significantly improved outcomes. Important clinical goals are more rapid identification of affected patients and accurate selection of antibiotics [15–17]. 

Aspiration pneumonitis following regurgitation of gastric contents under general anesthesia, although rare, occurring approximately 1 in every 3,000 general anesthetics, can result in devastating consequences. The mortality rate following aspiration of gastric contents is 1 in 71,000 general anesthetics [18]. Obese patients have typically been considered to have high-volume, low pH gastric contents that may lead to increased risk of aspiration pneumonitis, although these findings have not been confirmed in more recent studies [19–21]. 

Pulmonary function can be improved in intubated patients by applying CPAP or PEEP to help avoid atelectasis, improve oxygenation, and reduce CO_2_ [3]. BiPAP has been shown to significantly reduce pulmonary dysfunction following gastroplasty in obese patients and accelerates reestablishment of preoperative pulmonary function [22]. It would seem likely that this may be beneficial in morbidly obese patients following thoracic surgery as well. Thoracic epidural analgesia has been shown to significantly improve vital capacity in obese patients following midline laparotomy surgery over intravenous opioid analgesia alone and should be considered [23]. Della Rocca et al. reported thoracic epidurals to be superior to IV morphine in terms of analgesia, hospital length of stay, and postoperative complications, such as nausea and vomiting [24]. Paravertebral blocks have been shown to be superior in thoracotomies to thoracic epidural, with fewer complications, such as hypotension and postoperative nausea and vomiting. Although regional techniques of any kind may pose significant challenges in the morbidly obese, consideration should be given to improve postoperative pulmonary function and reduce complications. Of note, contraindications to thoracic epidural placement do not preclude paravertebral blocks as there appears to be a decreased risk of neurological injury [25]. Dexmedetomidine, an alpha 2 agonist with properties that have minimal effects on respiration, may be useful in these patients [26].

## 5. Cardiac Complications

Right ventricular dysfunction can be demonstrated by echocardiography in many obese patients, even if asymptomatic [27]. A great risk of pulmonary and systemic hypertension, related to increased blood volume and higher cardiac output, may exist in morbidly obese patients. Obesity cardiomyopathy with left- and right-heart failure secondary to eccentric right and left ventricular hypertrophy may result if these are present for a long period of time. The risk may be increased in the presence of long-standing obstructive sleep apnea [28]. 

Atrial fibrillation is a common complication in the obese patient, with risk increasing 4% per 1-unit increase in BMI [29]. In a study of 2588 patients undergoing noncardiac thoracic surgery, patients who developed atrial fibrillation had increased mean lengths of stay, higher mortality rates, and greater mean hospital charges. Significant variables associated with increased occurrence of atrial fibrillation were male sex, age, history of congestive heart failure, history of arrhythmias, history of peripheral vascular disease, resection of mediastinal tumor, lobectomy, bilobectomy, pneumonectomy, esophagectomy, and intraoperative transfusions [30]. Therapies targeted to reduce the incidence of atrial fibrillation in morbidly obese patients undergoing thoracic surgery seem warranted.

## 6. Other Postoperative Complications

The risk of thromboembolism is thought to be greater in morbidly obese patients [31]. Deep venous thrombosis and skin ulcerations are common in the morbidly obese patient. Varicose veins may occur, and lymphoedema may result [32]. Pulmonary embolism is a real risk for these patients, especially those with decreased mobility [33]. Morbidly obese patients have been shown to have a greater incidence of postoperative complications than normal-weight patients undergoing cardiac surgery [34]. Meticulous venous thromboembolism prophylaxis is necessary to decrease the incidence of these complications.

There is a strong correlation between obesity and diabetes mellitus (DM), type 2, in all ethnic populations. Obesity is attributed to be the cause in more than 80% of Type 2 DM [32]. Morbidly obese patients have a higher incidence of DM as the degree and duration of obesity, as well as distribution of body fat, increase the risk [35]. Close monitoring of postoperative glucose levels is recommended.

Although no studies have suggested a target glucose level postoperatively in patients who have undergone thoracic surgery, poor intraoperative control of glucose levels in patients undergoing cardiac surgery has been shown to worsen hospital outcomes [36]. However, a recently reported clinical trial of intensive insulin therapy (ACCORD) found a 3- to 4-fold increase in hypoglycemia which was associated with excess mortality. The risks increased in older patients, coexisting severe comorbidities, the presence of undiagnosed hypoglycemia, and the duration of diabetes and insulin therapy [37, 38]. It is likely that in morbidly obese patients with coexisting type 2 DM undergoing thoracic surgery the same outcomes may be expected. A recommended target minimum glucose level of 200 mg/dL has been suggested [35]. 

The changes in pharmacokinetics and pharmacodynamics caused by obesity provide further challenges in postoperative management of the morbidly obese patient. Organs involved in drug elimination (liver, kidney, etc.) may be affected, and drug administration is difficult, as most recommended doses are based on data obtained from individuals with normal weight. Use of total body weight instead of ideal body weight may result in an inadvertent overdose. Respiratory depression from excessive opioids has been previously discussed. Residual neuromuscular blockade from inappropriate dosing can be lethal. Reversal of neuromuscular blockade with neostigmine has been suggested in all morbidly obese patients, and close monitoring following extubation in the postanesthesia care unit is necessary [39]. 

Complications following anesthesia for thoracic surgery in morbidly obese patients may also include the same as for other patients, such as nerve injury due to inadequate padding and inappropriate positioning, perioperative myocardial ischemia or infarction, or untoward neurologic sequelae, such as stroke.

## 7. Conclusion

Although little has been published specifically related to morbidly obese patients undergoing thoracic surgery, the risk of postoperative complications is high, which can result in increased morbidity and mortality, increased length of stay, and increased costs of care. Anesthesia providers must be aware of these complications to prevent and manage these patients in the postoperative period. Clinical outcome studies specifically related to the morbidly obese patients are still lacking.
